# pepDESC: A Method for the Detection of Differentially Expressed Proteins for Mass Spectrometry-Based Single-Cell Proteomics Using Peptide-level Information

**DOI:** 10.1016/j.mcpro.2023.100583

**Published:** 2023-05-24

**Authors:** Yutong Zhang

**Affiliations:** 1 Collage of Chemistry and Molecular Engineering, Peking University, Beijing, China; 2 Beijing Advanced Innovation Center for Genomics, Peking University, Beijing, China; 3 Biomedical Pioneering Innovation Center, Peking University, Beijing, China

**Keywords:** single-cell proteomics, differential expression, label-free quantification, gene expression noise, peptide-level, statistics

## Abstract

Single-cell proteomics as an emerging field has exhibited potential in revealing cellular heterogeneity at the functional level. However, accurate interpretation of single-cell proteomics data suffers from challenges such as measurement noise, internal heterogeneity, and the limited sample size of label-free quantitative mass spectrometry. Herein, the author describes peptide-level differential expression analysis for single-cell proteomic (pepDESC), a method for detecting differentially expressed proteins using peptide-level information designed for label-free quantitative mass spectrometry-based single-cell proteomics. While, in this study, the author focuses on the heterogeneity among the limited number of samples, pepDESC is also applicable to regular-size proteomics data. By balancing proteome coverage and quantification accuracy using peptide quantification, pepDESC is demonstrated to be effective in real-world single-cell and spike-in benchmark datasets. By applying pepDESC to published single-mouse macrophage data, the author found a large fraction of differentially expressed proteins among three types of cells, which remarkably revealed distinct dynamics of different cellular functions responding to lipopolysaccharide stimulation.

## Highlights

• pepDESC performs differential expression analysis for single-cell proteomics data.
• pepDESC uses peptide-level quantification signals to improve analysis results.
• pepDESC performs well on low-input and regular-size benchmark datasets.
• pepDESC is compatible with different search engines and various workflows.
• pepDESC is available as an R package.

## Graphical Abstract

**Figure:**
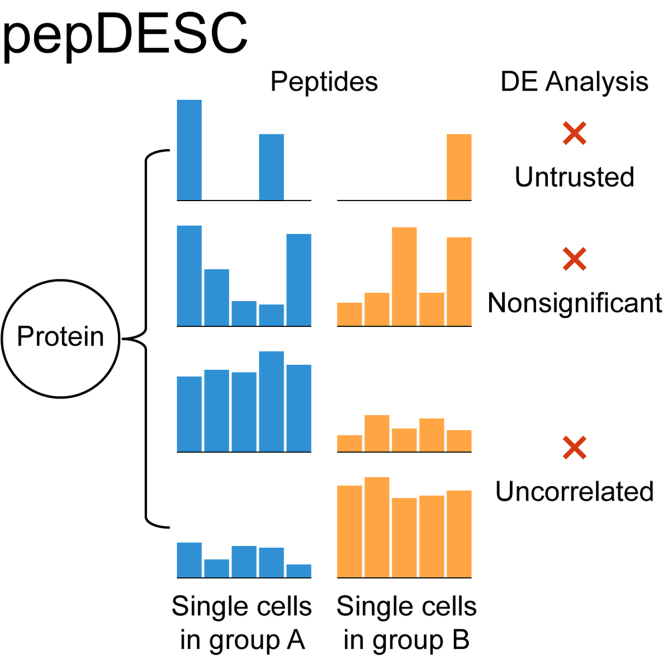

## In Brief

pepDESC is a statistical method built for differential expression analysis of single-cell mass spectrometry-based data. To overcome the difficulties caused by the small amount of input sample, pepDESC uses peptide-level quantification results to balance the proteome coverage and quantification accuracy. The application of pepDESC shows its superiority in benchmark datasets and real single-cell measurements. The use of pepDESC would improve the current implementation of single-cell proteomics measurements and boost our understanding of single-cell data at the functional level.

Single-cell proteomic measurements, which are capable of accessing cell identities at the functional level, have demonstrated the potential in providing insights into cellular activities and pathological mechanisms (1). Cutting-edge strategies for sample preparation (2, 3), chromatographic separation (4, 5) and database searching (6) designed for mass spectrometry (MS)-based single-cell proteomics have witnessed tremendous progress in recent years. However, attention on quantification accuracy in this domain remains insufficient. In fact, analyzing single-cell MS-based proteomics data, for example, to detect the differential expression of proteins between two cell types is quite challenging because of low signal-to-noise ratio. Besides the relatively high measurement noise, the existence of a large fraction of missing values hinders the accurate interpretation of data (1). Furthermore, it would be more difficult to analyze differentially expressed proteins with intrinsic heterogeneity within each cell type (7, 8). While label-free quantification has, to some extent, avoided the limitation due to the carrier proteome effect of isobaric-labeling quantification (9), the statistical confidence of single-cell proteomics data would be affected by the sample size (8), which is technically a major challenge of concomitant to label-free quantitative MS and an inevitable difficulty when it comes to scarce sample.

Methods designed for analyzing bulk proteomics data are not always suitable for single-cell proteomics data. For instance, in most cases, proteins with only one reliable peptide would be removed to avoid false identification (10, 11). However, applying such a stringent criterion to single-cell data would sacrifice proteome coverage when the quantified protein number is already very small. At the same time, although single-cell transcriptomics techniques have provided well-established measurement tools for differential expression analysis, they may not be the most optimal choices for proteomics analysis. This is because of the hierarchical structure of bottom-up MS-based proteomics data, where protein abundances rely on aggregating peptide-level or peptide spectrum matches (PSM)-level information (12).

Recently, it has been acknowledged that using peptide-level information is promising in proteomics studies (13). Several methods for analyzing differentially expressed proteins at the peptide level have already been proposed (14, 15). However, whether existing methods are applicable to single-cell proteomics data is still in question, and suitable statistical tools for single-cell proteomics data are still required.

Herein, the author introduces a method, PEPtide-level Differential Expression analysis for Single-Cell proteomic (pepDESC), for analyzing differential expression scores at the peptide level according to the nature of single-cell proteomics data. This tool directly uses peptide-level results and is compatible with various search engines and is available as an R package. Several commonly used statistical methods and peptide-based methods were used for comparison to state the strengths of pepDESC in low-input MS data as well as regular MS data. Moreover, the author applied pepDESC to a published single-cell experiment to demonstrate its performance in real-world data.

## Experimental Procedures

### Experimental Design and Statistical Rationale

Three datasets were used to validate the performance of pepDESC, namely, D1 (a mixed single-cell data), D2 (a low-input spike-in data), and D3 (a regular-size spike-in data). The dataset D1 contains ten biological replicates in each sample group. The spike-in datasets D2 and D3 contain seven and four technical replicates in each sample group, respectively. The design of group size considered the current sample size of label-free quantitative MS-based proteomics experiment (3, 16, 17). The sample preparation of dataset D2, the database searching of the three datasets, and the design of the dataset D1 are explicated herein with detailed information presented below.

### Sample Preparation of D2 Low-Input Spike-In Dataset

The *Escherichia coli* DH5α cell pellet was lysed by sonication using a Covaris M220 in a buffer containing 8 M urea and 100 mM TEAB. The protein amount was measured using a micro BCA protein assay kit (23235; ThermoFisher). The sample was reduced using a final concentration of 0.25 M DTT and alkylated with a final concentration of 8 mM IAA. The trypsin (V5280; Promega) was then added to 1:50 enzyme-to-protein ratio in 100 mM TEAB. The sample was acidified with a final concentration of 1% TFA before being loaded into the SPE micro-column. The peptides were eluted after two times of washing and were dried in a SpeedVac. Finally, *E. coli* proteins were reconstituted with 0.1% TFA and 1% ACN buffer. The HeLa digest (88329, ThermoFisher) was reconstituted with 0.1% TFA and 1% ACN. The *E. coli* digest and HeLa digest were mixed at the total mass ratio of 94:6 or 97:3.

### LC-MS/MS Analysis of Dataset D2 Low-Input Spike-In Dataset

For each sample group, seven technical replicates of measurements were conducted to mimic the sample size of current label-free single-cell proteomics data. First, 120 pg of digests were injected and separated using a commercial chromatography column (Aurora, IonOpticks) by a nanoflow liquid chromatography (Ultimate 3000 RSLCnano, ThermoFisher) with a flow rate of 100 nl/min. Mobile phase A was 0.1% FA in 2% acetonitrile, while mobile phase B was 0.1% FA in 80%acetonitrile. The 70-min LC gradient was as follows: 5% to 6.2% B for 2 min, 6.2% to 31.2% B for 40 min, 31.2% to 42.5% B for 16 min, 42.5% to 99% B for 5 min, and then isocratic at 99% B for 10 min. Label-free quantification was performed using a tribrid mass spectrometer (Orbitrap Eclipse, ThermoFisher) with an ion mobility interface (FAIMS Pro, ThermoFisher). The FAMIS compensation voltages of −55 V and −70 V were used with a cycle time of 1 s. The MS spectra were collected by an Orbitrap analyzer and the MS2 spectra were collected by a linear ion trap analyzer with a max ion injection time of 200 ms.

### Database Searching of the datasets D1, D2, and D3

The database searching was conducted by Proteome Discoverer 2.4 (ThermoFisher) using Sequest. For the mixed single-cell dataset, the 293T cell sample and the mouse oocyte sample were searched separately against the UniProt human protein database (20,286 entries, downloaded on April 14, 2020) and UniProt mouse database (17,015 entries, downloaded on downloaded on July 31, 2020) respectively. Datasets D2 and D3 were searched against the UniProt human protein database (20,286 entries, downloaded on April 14, 2020) with the UniProt *E. coli* database (4349 entries, downloaded on July 15, 2019) concatenated. An in-house curated contamination database by Proteome Discoverer 2.4 (ThermoFisher) containing 284 entries was also included in each analysis. The carbamidomethylation on cysteine residues was set as a static modification. Dynamic modifications included the acetylation on N-terminals, the methylation loss on N-terminals, and the oxidation on methionine residues. The mass tolerance for precursor ions was 10 ppm while the mass tolerance for fragment ions was 0.6 Da. At most, two tryptic miss-cleavages were allowed. PSMs and proteins were both filtered at 1% false discovery rate. The protein-level search results are shown in Supplementary File S1, and correspondingly, the peptide-level search results are shown in Supplementary File S2.

For D1, the human dataset of 20 293T cells contains 1409 high-confidence master proteins and 7694 high-confidence peptides, while the mouse dataset of 20 mouse oocytes contains 3800 high-confidence master proteins and 32,098 high-confidence peptides.

For D2, the result contains 1409 high-confidence master proteins and 5726 high-confidence peptides. For D3, the result contains 4967 high-confidence master proteins and 29,117 high-confidence peptides.

### Design of D1 Mixed Single-Cell Benchmark Dataset

To design a benchmark dataset with certain different proteins while retaining the characteristic of single-cell proteomics data, a few modifications were made to dataset D1. First, among the 1409 human proteins and the 3800 mouse proteins identified by the search engine, 200 proteins in 20 293T cells as well as 1000 proteins in 20 mouse oocytes were randomly selected while the remaining proteins were removed. Next, the human cells with 200 proteins and the mouse cells with 1000 proteins were combined into 20 samples, and each has the expression of 1200 proteins from a particular 293T cell and a particular mouse oocyte. To make a certain differentially expressed protein set, the abundances of 200 human proteins in ten samples were numerically halved. That is, the dataset D1 contains two sample groups of ten biological replicates with 200 differentially expressed proteins (marked as human proteins) and 1000 stable proteins (marked as mouse proteins). The dataset D1 is shown in the Supplementary File S3.

### Applying Different Statistical Methods to Datasets D1, D2, and D3

In this paragraph, the abundance of peptides or proteins is denoted with the letter X, where X_N_ is the abundance of a peptide or protein in the sample group N.

### Statistical Methods Based on Protein Abundances

High-confidence master proteins were normalized by the median value of each sample group, with missing values filled with 0 s. These data were used for Student’s *t* test, Wilcoxon test, and the Limma method. The statistics for ordinary Student’s *t* test are as follows:

(1) $$s_{p}=\sqrt{\frac{s_{X1^{2}}+s_{X2^{2}}}{2}}$$

(2) $$t=\frac{\bar{X_{1}}-\bar{X_{2}}}{s_{p}\sqrt{\frac{1}{n_{1}}=\frac{1}{n_{2}}}}$$

n_1_ and n_2_ are the sample sizes of the two groups of samples.

The two-sided Wilcoxon test (also known as Mann–Whitney Wilcoxon test) was based on the ranks of each data in two sample groups. The statistics denoted as U for two sample groups are:

(3) $$U_{1}=n_{1}n_{2}+\frac{n_{1}\left(n_{1}+1\right)}{2}-R_{1}$$

(4) $$U_{2}=n_{1}n_{2}+\frac{n_{2}\left(n_{1}+1\right)}{2}-R_{2}$$

n_1_ and n_2_ are the sample sizes of the two groups of samples. R_1_ and R_2_ are the sums of ranks of the two groups of samples.

Limma was performed based on the empirical Bayesian prior variance:

(5) $$t=\frac{\bar{X_{1}}-\bar{X_{2}}}{S_{posterior}\sqrt{\frac{1}{n_{1}}=\frac{1}{n_{2}}}}$$

s_posterior_ was derived by the eBayes() function from the Limma package.

Student’s *t* test was accomplished by function t.test() from “stats” package. Wilcoxon test was accomplished by wilcox.test() from “stats” package. Limma was accomplished by the “Limma” package.

### Statistical Methods Based on Peptide Abundances

For high-confidence unique peptides, the missing values were filled with 0 s. These data were used for the PECA, DeqMS, and pepDESC.

The DeqMS only works for peptides whose minimum is greater than zero. Normalization of each group sample by the median value was done after applying log-transformation of the data. The DeqMS uses the core method of Limma to the peptide abundances and adjusts the statistics with peptide counts, which is accomplished by the spectraCounteBayes() function in the “DEqMS” package (14).

PECA was performed by a one-line function PECA() from the “PECA” package. The PECA method simply calculates the statistics of Student’s *t* test for all the peptides from a protein to discover differentially expressed proteins. Normalization in PECA was included for the analysis of datasets D2 and D3. No normalization was used for dataset D1 since group-wise normalization is not compatible with this method (15).

The first step of pepDESC was to filter out the contamination peptides from the contamination protein database, peptides with missing values over 60%, or a customized threshold, of cells and peptides with peaks identical to contamination peptides (retention time difference <0.05 and m/z difference <2). Normalization was done after removing outlier samples. pepDESC adopt a median normalization by default unless with user-defined settings or any sample has over 50% missing values, where a mean normalization would be adopted. The mathematical details of DE-scores, which mark the confidence of a changing protein or a peptide, are illustrated with the following equations.

When the first sample group has M cells while the second sample group has N cells, denote the abundance of peptide *i* in different cells to be $\left\{X_{i,1},X_{i,2},\ldots ,X_{i,m},\ldots ,X_{i,M}\right\}$ and $\left\{X_{i,1},X_{i,2},\ldots ,X_{i,n},\ldots ,X_{i,N}\right\}$.

The adjusted DE-score of a peptide is derived based on the pairwise ratio of a peptide abundance between two sample groups. Peptide DE-score whose absolute value was found to be higher than 1.5 was set as 1.5 at Equation (6).

(6) $${\text{DE}-\text{Score}}_{i}=log_{2}\left(median\left\{\frac{X_{i,m}}{X_{i,n}}m\in M,n\in N\right\}\right)$$

(7) $${\text{DE}-\text{Score}}_{i,\text{adjusted}}={\text{DE}-\text{Score}}_{i}*W_{i}\text{.}$$

Where the second term denotes the fidelity of a peptide by its expression level and the correlation between other peptides of this protein (Equation (8)). The third term describes if the peptide is significantly different between sample groups.

(8) $$W_{i}^{\prime }=\bar{X_{i}}*\sum_{j\in I}r_{ij}$$

r_ij_ describes the Pearson correlation coefficient of two peptides if they were positively correlated:

(9) $$r_{ij}=\left\{ \begin{array}{cc} \frac{\bar{\left(X_{i}X_{j}\right)}-\bar{X_{i}}\bar{X_{j}}}{\sqrt{\bar{X_{i}^{2}}-{\bar{X_{i}}}^{2}}\sqrt{\overset{‾}{X_{j}^{2}}-{\bar{X_{j}}}^{2}}} & \text{if}\frac{\bar{\left(X_{i}X_{j}\right)}-\bar{X_{i}}\bar{X_{j}}}{\sqrt{\bar{X_{i}^{2}}-{\bar{X_{i}}}^{2}}\sqrt{\bar{X_{j}^{2}}-{\bar{X_{j}}}^{2}}}>0\\ 0 & \text{if}\frac{\bar{\left(X_{i}X_{j}\right)}-\bar{X_{i}}\bar{X_{j}}}{\sqrt{\bar{X_{i}^{2}}-{\bar{X_{i}}}^{2}}\sqrt{\bar{X_{j}^{2}}-{\bar{X_{j}}}^{2}}}\leq 0 \end{array}\right.$$

The weight coefficients were then normalized among peptides:

(10) $$W_{i}=\frac{W_{i}^{\prime }}{\sum_{i\in I}W_{i}^{\prime }}$$

Finally, the DE-score of a protein is derived based on all the adjusted DE-scores of the belonging peptides:

(11) $$\text{DE}-\text{Score}=\sum_{i\in I}\left({\text{DE}-\text{Score}}_{i,\text{adjusted}}*\delta_{i}\right)$$

(12) $$\delta_{i}=\left\{ \begin{array}{c} 1\;\text{if}\;p_{i}<0.05\\ 0\;\text{if}\;p_{i}\geq 0.05 \end{array}\right.$$

When a protein is quantified based on a single peptide, the DE-score goes with Equation (13)

(13) $$\text{DE}-\text{score}=\left\{ \begin{array}{c} \log\;2\left(median\left(\left\{\frac{X_{i,m}}{X_{i,n}}m\in M,n\in N\right\}\right)\right)\;\text{if}\;p_{i}<0.05\\ 0\;\text{if}\;p_{i}\geq 0.05 \end{array}\right.$$

In the Equations (12) and (13), the p_i_ denotes the possibility of taking the null hypothesis in the Wilcoxon test. The maximum allowed *p*-value was set as 0.05 by default, yet a customized setting is allowed with pepDESC. All the adjustable parameters mentioned above were set as default values for analysis of D1, D2, and D3.

### Evaluating the Performance of Different Statistical Methods

The precision–recall curves were plotted using the R package “ROCR”. The number of true positive discoveries refers to the number of *E. coli* proteins (for D2 and D3) or the human proteins (for D1) identified as varying in each statistical method. The precision of the results refers to the ratio of true positive discoveries to total positive discoveries.

### Applying pepDESC to Single-Cell Proteomics Result of Mouse Macrophages

The single-cell proteomics data of 164 mouse macrophages was downloaded *via* ProteomeXchange on March 22, 2022 (3). The peptide result containing 31,391 unique peptides was used as the input data for pepDESC. The protein result containing 1979 proteins was used as a reference. The application of pepDESC contains two separate analyses, the analysis between the control group (abbreviated as CON) and 24 h stimulation group (abbreviated as LPS24), and the analysis between LPS24 and the 48 h stimulation group (abbreviated as LPS48). Contamination peptides denoted by the search result and the peptides with more than 80% missing values were removed in each analysis, and outlier samples were also removed in each analysis. Mean normalizations in each group were applied since a high fraction of missing values existed in several samples. Peptides whose accession starts with “CON” or “REV” were set as contamination peptides.

Pathway enrichment was conducted using the “Analyse gene list” function in the Reactome knowledgebase resource (18), release 83.

## Results

### Building the Peptide-Level Differential Expression Analysis Method Based on the Nature of Single-Cell Proteomics Data

**Fig. 1:**
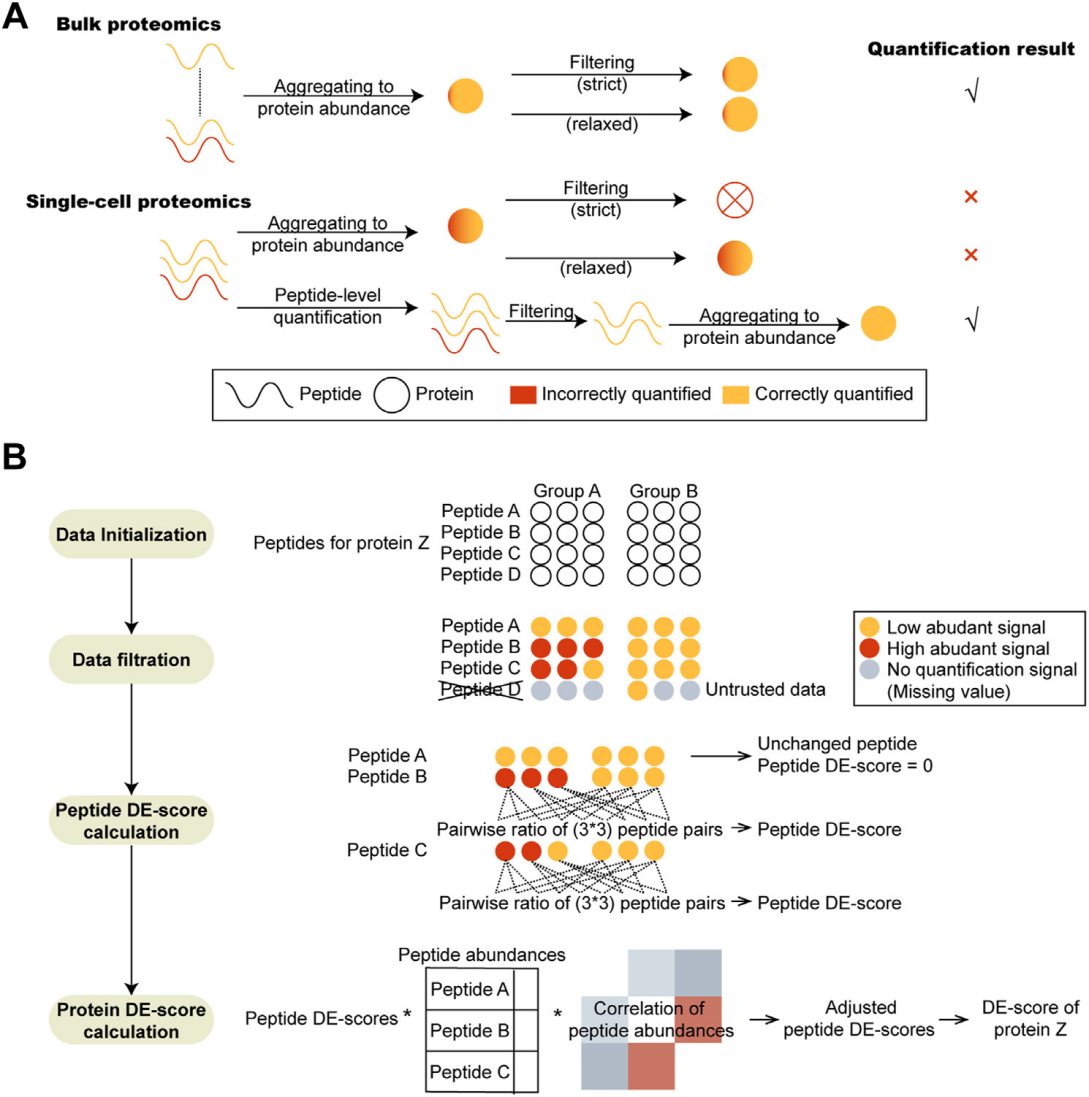
**Design of pepDESC, a method to discover differential expression of proteome at peptide-level for single-cell proteomicsa.***A*, One reason to use peptide-level information for single-cell proteomics data. The lines show the quantification results of peptides while the circles show the quantification results of proteins. The *red color* indicates that a peptide or a protein is incorrectly quantified while the *yellow color* indicates that a peptide or a protein is correctly quantified. In bulk proteomics (*top*), a protein is usually quantified by a number of peptides, where the accuracy of quantification result is merely affected by one falsely quantified peptide. While in single-cell proteomics (*bottom*), many proteins are quantified by a limited number of peptides, one falsely quantified peptide will bring non-negligible effect to the final result, resulting in a sacrifice in coverage or accuracy. In this case, processing data at the peptide-level has the potential to remove falsely quantified peptides to assure the accuracy of the statistical result. *B*, The workflow of pepDESC is illustrated by protein Z, an example protein with four quantified peptides. pepDESC is composed of four steps, data initialization, data filtration, peptide DE-score calculation, and protein DE-score calculation. In the first step, information regarding the four peptides is extracted from the input search result. In the second step, “untrusted” data, which in this case refers to the peptide D, which had missing values in five samples, is identified and removed. In the third step, peptide DE-scores are calculated based on the pairwise ratio between peptide abundances in two types of samples. However, since the expression of peptide A is not significantly different between the two groups, the peptide DE-score of peptide A was 0. Finally, protein DE-scores were derived based on the adjusted peptide DE-scores, considering the expression level and correlations of the three peptides.

An optimal method for single-cell proteomics analysis needs to balance the quantification accuracy and proteome coverage. While relaxed data filtration negatively impacts the reliability of quantification results, strict data filtration challenges the depth of data. Therefore, processing the data at the peptide level would theoretically improve the overall performance (Fig. 1*A*). Based on the quantification results of single-cell MS measurements generated by search engine, the author built pepDESC, by which a DE-score quantifies the result of differential expression analysis. pepDESC includes three major steps: data filtration, peptide DE-score calculation, and protein DE-score calculation (Fig. 1*B*).

At the beginning, two types of “untrusted” data need to be removed. The first type of “untrusted” data refers to contamination peptides assigned by the search engine and peptides that are highly suspected to be contaminated (19). The second type of “untrusted” data contains too many missing values, which could be misleading during the downstream analysis (20).

The difference in each peptide was calculated based on the pairwise ratio between each sample pair from two different sample groups, which was then defined as the peptide DE-score. A higher peptide DE score means a higher magnitude of differences. However, owing to the stochasticity in gene expression, the single-cell proteomics data are naturally noisier than bulk proteomics, thereby leading to internal heterogeneity among identical cells (7). Therefore, the author further measured the statistical significance of the abundance differences to identify DE scores that were merely caused by internal heterogeneity.

When aggregated to protein DE scores, the credibility of the peptide DE scores should be evaluated again. Falsely identified peptides could occur during database searching (10) and match-between-runs algorithm (21). Traditionally, when using the summation of the peptide abundances as the abundance of a protein, the weight coefficient of each peptide is solely dependent on the expression level. However, in single-cell proteomics, where peptide number is limited for each protein, the impact of falsely quantified high-abundant peptides needs to be further reduced. As a protein is usually quantified by several different peptides, whether a peptide truthfully indicates the abundance of the protein or not can be testified by each other. Thus, the reliability of peptide DE scores could be informed by the correlation of the peptides belonging to the same protein. Therefore, the author calculated the adjusted peptide DE-scores considering the abundances as well as the pairwise abundance correlations and determined the protein DE scores considering all adjusted DE scores of belonging peptides, as described in the Experimental procedures section.

### Performance on Mixed Single-Cell Data Reveals the Strengths of pepDESC

The author first tested pepDESC using mixed single-cell data, addressed as dataset D1 (Supplementary File S3), which was adapted from a label-free single-cell proteomics experiment on homogenous 293T human cells and mouse MII oocytes (22). This dataset comprises 20 samples, each containing 200 proteins from a human 293T cell and 1000 proteins from a mouse oocyte cell. Ten of them contain 1000 proteins from ten different mouse oocytes and 200 proteins from ten different human 293T cells, while the other ten samples contain 1000 proteins from ten different mouse oocytes and 200 proteins from ten different half-293T cells, as described in the Experimental procedures section. This benchmark dataset represented a single-cell proteomics dataset with internal heterogeneity and external differences. In this scenario, an ideal method should identify all 200 human proteins and no mouse protein as differentially expressed proteins.

To state the performance of pepDESC, the author roughly compared the protein DE scores with the result of the commonly used Student’s *t* test. pepDESC found 140 differentially expressed human proteins with 33 falsely identified mouse proteins (absolute value of protein DE-score >0.3) while Student’s *t* test found 118 differentially expressed human proteins and 48 falsely identified mouse proteins (*p* value < 0.05). By studying the proteins that had different results with the two methods, it could be found that some error-prone steps in the traditional protein quantification method could be circumvented using pepDESC.

**Fig. 2:**
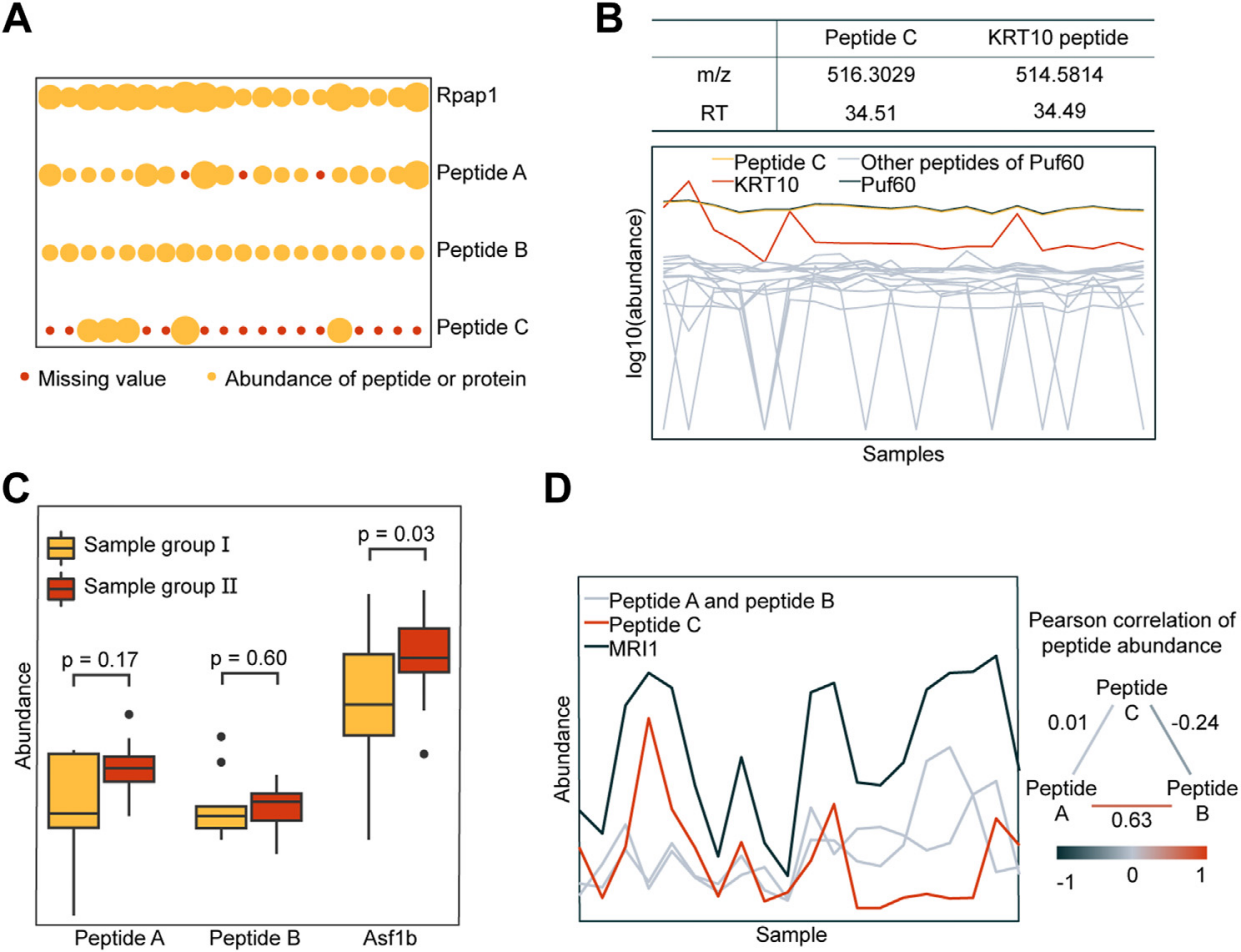
**Comparison between pepDESC and Student’s *t* test with dataset D1.** Student’s *t* test identified a changing protein when *p*-value is lower than 0.05, while pepDESC identified a changing protein when the absolute value of DE-score is over 0.3. Human proteins were theoretically changing while mouse proteins should be stable proteins. *A*, Bubble plot for mouse Rpap1 protein and peptides. The size of circles stands for the abundance of corresponding protein or peptide. A *red circle* indicates a missing value. The result of Rpap1 for Student’s *t* test was incorrect (changed, *p* value = 0.01). pepDESC removed the peptide C during filtration and the result of Rpap1 for pepDESC was correct (unchanged, DE-score = 0.26). *B*, Peak feature chart and line plot for mouse Puf60 protein and peptides. The chart (*top*) shows the similarity of the peak features of peptide C and a KRT10 peptide (contaminant peptide). At the same time, peptide C had similar expression level with KRT10 (*down*). The result of Puf60 for Student’s *t* test was incorrect (changed, *p* value = 0.03). pepDESC removed the peptide C during filtration and the result of Puf60 for pepDESC was correct (unchanged, DE-score = 0.11). *C*, Boxplot for mouse Asf1b protein and peptides in two sample groups, with *p*-values for Student’s *t* test of peptide and protein abundances. The result of Asf1b for Student’s *t* test was incorrect (changed, *p* value = 0.03). The result of Asf1b for pepDESC was correct (unchanged, DE-score = 0). *D*, Line plot for human MRI1 protein and peptides abundances (*left*) with the Pearson correlation of peptide abundances (*right*). The result of MRI1 for Student’s *t* test was incorrect (unchanged, *p*-value = 0.16). The result of MRI1 for pepDESC was correct (changed, DE-score = 1.13).

For real-world single-cell proteomics data, the most common challenge during differential expression analysis is to deal with the large fraction of missing values. In 140 falsely identified proteins in Student’s *t* test, 54 proteins were affected by peptides with missing values over 60%. Although generally speaking, summing up the abundances over several peptides could alleviate this problem, for proteins with limited quantified peptides, the existence of these inaccurate quantified peptides would affect the quantitative results of the proteins. For example, one peptide (peptide C in Fig. 2*A*) of Rpap1 had a large fraction of missing values, which would mislead the protein-level measurement if not removed in advance (Fig. 2*A*). High-abundance peptide might also need to be removed. For mouse protein Puf60, its abundance was highly dominated by a peptide (peptide C in Fig. 2*B*) that was highly likely to be a contamination signal. pepDESC found this misidentified peptide as it had similar features and similar expression level with a contaminant peptide (Fig. 2*B*). This misidentification might be ascribed to a misassignment during match between runs, which merely affects bulk experiments where the true signals of samples are much higher than contamination signals.

A common mistake reported when using Student’s *t* test to analyze single-cell data was caused by the accumulated noises from “unchanged peptides”. In 13 out of 48 falsely positive proteins, no peptide was significantly different between the two sample groups. For example, neither peptide of Asf1b was considered changing (Student’s *t* test *p* value > 0.1). Yet, the numerical summation of the peptides, which the abundance of Asf1b protein, between two groups of samples, was significantly different (Student’s *t* test *p* value = 0.03; Fig. 2*C*).

Furthermore, the credibility of peptide DE-score was further tested in pepDESC. In the case of human protein MRI1, the most abundant peptide (peptide C in Fig. 2*C*) correlated poorly with the other two peptides. If a traditional method was adopted and the sum of the peptide abundances was used as the protein abundance, this highly abundant peptide would affect the final result and lead to a wrong conclusion (Fig. 2*D*).

As evidenced by the result, processing data at the peptide level does improve the quantification performance for this benchmark dataset, which is based on real-world single-cell proteomics measurement.

### Comparison of Different Differential Expression Detection Methods

To assess the performance of pepDESC compared with other statistical tools, the author evaluated several approaches including Student’s *t* test, Wilcoxon test, and widely used “Limma” (23) using three label-free proteomics benchmark datasets. The author also compared pepDESC with peptide-level quantification methods DEqMS and PECA.

**Fig. 3:**
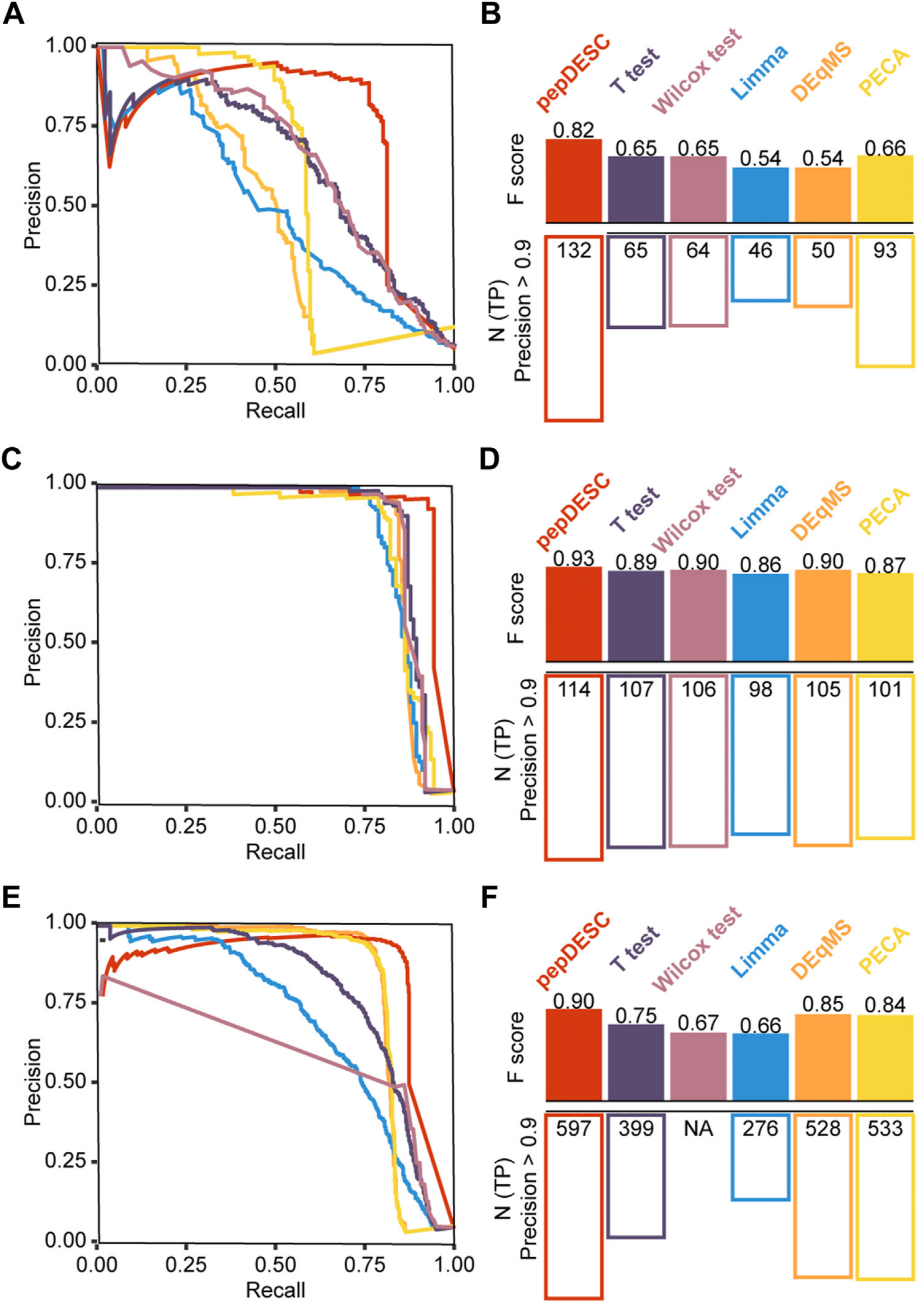
**Performance of different methods on datasets D1, D2, and D3.** The precision recall curves in (*A*), (*C*), and (*E*), where different colors stand for the performance of different methods. The bar plots in *B*, *D*, and *F* show the F-scores corresponding to each method (*top*) and the maximum number of true positive identification when the overall precision was found to be bigger than 0.9. Student’s *t* test was abbreviated as “*t* test”. The color of the precision–recall curves corresponds to the color in the bar plots. *A* and *B*, Performance of different methods on dataset D1. *C* and *D*, Performance of different methods on dataset D2. *E* and *F*, Performance of different methods on dataset D3.

First, the performance of all these methods on the dataset D1 was determined (Fig. 3*A*). As evident from the precision–recall curves, pepDESC attained the highest F-score and outperformed other methods. Meanwhile, it had the largest correct identification with precision over 0.9.

Next, a series of spike-in samples with different compositions of HeLa digest and *E. coli* digest were collected. Each sample group of this benchmark dataset, addressed as dataset D2, contains seven technical replicates. The two groups of samples contained 3% or 6% of *E. coli* protein and 97% or 93% of human protein, respectively. The amount of peptides loaded into the MS was around 120 pg to imitate the protein contents of a single cell. In this case, ideal quantification results should show that the *E. coli* proteins change around two folds, and the human proteins remain constant. Still, the precision–recall curves and the F-scores confirmed the superiority of pepDESC, and it can also identify more changing proteins when the precision is higher than 0.9 (Fig. 3*B*).

Furthermore, all the methods were applied to a published benchmark dataset for regular proteomics (24), addressed as dataset D3. This spike-in dataset contains four samples with 3% *E. coli* proteins in human proteins and four samples with 6% *E. coli* proteins in human proteins. Although all the tested methods obviously showed better performance compared to results for low-input data, as shown by Figure 3*C*, pepDESC outperformed other methods as well for the regular-size proteomic measurements.

Besides the high performance of pepDESC, it could also be noticed that through the analysis of three independent benchmark datasets, peptide-level quantification methods pepDESC, PECA, and DEqMS all showed satisfactory performance. DEqMS yielded the second-best result in the two spike-in datasets but was not a very ideal choice for dataset D1, as it was unfriendly with missing values. PECA outperformed other methods except for pepDESC in dataset D1. This result illustrates the superiority of peptide-based methods in studying proteomics data.

### Applying pepDESC to a Single-Mouse Macrophages Proteome Data Reveals Distinct Dynamics of Different Cellular Functions Replying to LPS Stimulation

To demonstrate the practicability of pepDESC in real-world single-cell proteomics data, it was applied to a published MaxQuant search result of single-mouse macrophage proteomics measurements (3). This dataset describes the single-cell proteome of 56 controlled cells, 56 24 h LPS-treated cells (abbreviated as LPS24), and 52 48 h LPS-treated cells (abbreviated as LPS48). The search result contains 1727 proteins with a large fraction of missing values. More specifically, there are 1264 missing values on an average for 1 cell, and only 383 proteins have expression over half of the cells. To tackle the problem caused by the missing values, the original work applied data imputation, despite a distortion of data (25), before differential expression analysis using ANOVA. The author wondered whether the use of pepDESC, which is more tolerant of missing values, could boost the performance without losing the fidelity of the original data. With the peptide quantitative information of the published search result, pepDESC applied two independent rounds of comparison between consecutive periods of time. A total of 452 changing proteins, 323 in the first 24 h and 271 in the second 24 h, were found to be changing with LPS stimulation among the 630 proteins in the pepDESC result (Supplementary File S4).

**Fig. 4:**
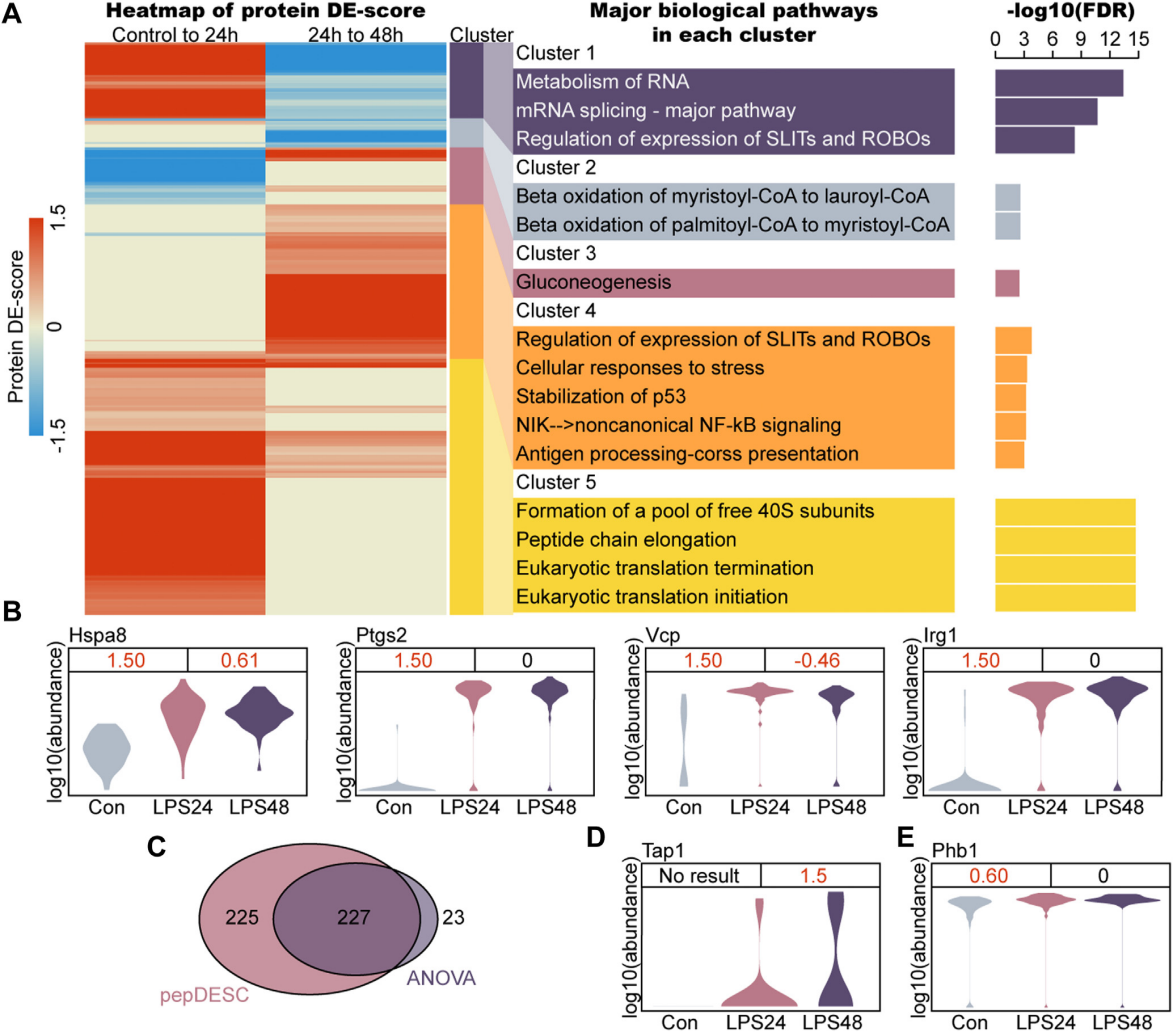
**Proteome dynamics for single-macrophages responding to LPS stimulation discovered by pepDESC.** The ANOVA result and the protein abundances were adapted from (3). *A*, Heatmap of protein DE-scores for 452 changing proteins (protein DE-score >0.3) during the first and the second 24 h after LPS stimulation (*left*). A positive DE-score means the protein has been upregulated during the period and the vice versa. 452 proteins were grouped into five clusters according to the protein DE-scores. Typical Reactome pathway enrichment terms (FDR <0.01) of each cluster is depicted in the middle. The FDR of each term is depicted in the bar plot (*right*). *B*, Dynamics of marker proteins found in the ANOVA result could also be found by pepDESC. The violin plot shows the log-transformed protein abundances from the protein level search result (with no imputation). The protein DE-scores of the two consecutive periods of time are shown on the *top*, where a changing protein is denoted with the red color. *C*, Venn plot of identified changing protein using pepDESC (*pink*) or using ANOVA (*purple*). *D* and *E*, Dynamics of newly identified marker protein using pepDESC. The violin plot shows the log-transformed protein abundances from the protein level search result (with no imputation). The protein DE-scores of the two consecutive periods of time are shown on the *top*, where a changing protein is denoted with the *red color*.

With protein DE-scores indicating the dynamics of the proteome, it could be found that various biological functions responded differently to LPS stimulation. The 452 changing proteins were grouped into five clusters, and each showed distinct dynamics and was involved in different biological pathways (18). As depicted in Figure 4*A*, proteins related to gene expression and protein translation in clusters 1 and 5 were upregulated in the first 24 h, whereas only the abundance of mRNA splicing proteins in cluster 1 dropped on the second day. A rise in stimulant-responding proteins and immunologically responding proteins primarily happens on the second day, as shown in cluster 4. Clusters 2 and Cluster 3 confirmed a change in the metabolism of LPS-stimulated macrophages (26).

Key regulators mentioned in the original work were also found by pepDESC (Fig. 4*B*). At the same time, most changing proteins found by ANOVA analysis could also be found with the new method (Fig. 4*C*), indicating a good overlap between the two methods. pepDESC additionally identified more marker proteins including the inflammatory signaling protein Tap1 (Fig. 4*D*), which is involved in antigen presentation *via* MHC class I (27) as well as the transcription regulator Phb1, which played a role in immunometabolism of macrophages (Fig. 4*E*) (28).

In summary, pepDESC uncovered a system-wide picture of proteome responding to LPS stimulation according to a specific time order. Usage of the new method successfully discovered a large number of differentially expressed proteins and offered an insightful interpretation of functional dynamics. pepDESC has been demonstrated to be a practical tool for real-world single-cell proteomics data that does not require imputation, which generally affects the data fidelity.

## Discussion

Single-cell proteomics data based on label-free quantitative MS has three main characteristics, namely, high measurement noise, internal heterogeneity, and limited sample size. To gain insights into such complicated data, there are two main concerns for statistical methods, which are proteome coverage and quantification accuracy. Carefully weighing the depth and the accuracy is crucial when dealing with single-cell proteomics data. Although various tools could be used to boost performance, such as data imputation, improper methods may reduce data fidelity and mask the intrinsic nature of single cells (25). Based on these considerations, the author developed a method, pepDESC, for single-cell proteomics discovery.

To demonstrate the performance of pepDESC, which uses the peptide-level information to discover differentially expressed proteins between 2 cell populations, three datasets were used for evaluation, including a mixed single-cell dataset (dataset D1), a low-input spike-in dataset (dataset D2), and a published regular spike-in dataset (dataset D3). Although it was clear that the performance of tested methods varied among different datasets, the advantage of pepDESC was evident despite limited sample size (D1, D2, and D3), internal heterogeneity (D1), and low quantification signal (D1 and D2). Compared with widely used protein-level methods, pepDESC evaluates the differences in data with the appreciation of the nature of single-cell data at a higher resolution. Although peptide-level methods PECA and DEqMS yielded good results as well, the stringent filtering condition dampened the performance in low-input data. Therefore, the choice of analysis tool should take both the feature of the data and the purpose of the analysis into consideration. As for the mouse macrophages data described in this work, although some key proteins like cytokines could not be detected as a result of their low copy numbers and the limited detectability of current single-cell MS, marker proteins could be discovered with effective analysis tools, like Tap1 and Phb1, which play critical roles during immunological response. To be specific, TAP1 would import the endogenous peptides into the endoplasmic reticulum after LPS stimulation, in order to present exogenous antigens *via* MHC class I molecules and activate CD8+ T cells, while the increased expression of Phb1 during the early stage of LPS stimulation regulates the orchestrating of the cytokine production. The discovery of the noisy data with a noneligible fraction of missing values greatly depended on the choice of the statistical method.

The design of pepDESC was to make it applicable to different quantification results, bulk or single-cell, using different search engines. In the current implementation, the slowest step to measure differentially expressed protein among around 100 cells only took a minute or less on personal computers, which makes pepDESC an effective tool even with larger cohorts. At the same time, to make this method compatible with various data, all the steps were sectioned allowing for customized workflows. Moreover, several parameters could also be adjusted based on the nature of the data.

The author hopes that this well-designed novel statistical tool would be widely used to improve the performance of MS-based single-cell proteomics technique. There is no doubt that more significant discoveries could be found with functional-level measurement at single-cell resolution.

## Data Availability

The raw data of dataset D3 is available on ProteomeXchange Consortinum *via* the PRIDE partner repository (identifier PXD003881). The raw data of single-mouse macrophages is available on ProteomeXchange Consortium *via* the MassIVE partner repository (identifier MSV000085937).

The raw data of D1 and D2 as well as the search engine result of D1, D2, and D3 are now private and have been deposited to MassIVE (https://doi.org/10.25345/C5Q814X3C).

The source data of pepDESC was available from Github (https://github.com/dionezhang/pepDESC).

## Supplemental data

.This article contains supplemental data.

## Conflict of interest

The authors declare no competing interests.
